# Sulphonamide derivatives with a triazine core as novel inducers of apoptosis and pyroptosis in glioblastoma multiforme cells

**DOI:** 10.1080/14756366.2025.2594158

**Published:** 2025-12-04

**Authors:** Olga Klaudia Szewczyk-Roszczenko, Katarzyna Kotwica-Mojzych, Piotr Roszczenko, Mariusz Mojzych, Adolfo Rivero-Müller, Karolina Czapla, Alicja Przybyszewska-Podstawka, Krzysztof Bielawski, Robert Czarnomysy

**Affiliations:** a Department of Synthesis and Technology of Drugs, Medical University of Białystok, Białystok, Poland; b Department of Histology, Embriology and Cytophisiology, Medical University of Lublin, ul. Radziwiłłowska 11 (Collegium Medicum), Lublin, Poland; c Department of Biotechnology, Medical University of Białystok, Białystok, Poland; d Collegium Medicum, The Mazovian Academy in Plock, Płock, Poland; e Department of Biochemistry and Molecular Biology, Medical University of Lublin, Lublin, Poland

**Keywords:** Glioblastoma multiforme, triazine sulphonamides, apoptosis, pyroptosis

## Abstract

Glioblastoma multiforme (GBM) is a highly aggressive brain tumour with few effective treatment options. This study evaluated two novel triazine-based sulphonamides, MM118 and MM119, for their anticancer effects on GBM cells. *In vitro* cell viability assays showed that both compounds were highly potent, killing GBM cells at low-micromolar concentrations. They induced apoptosis in cancer cells, evidenced by Annexin V/PI staining and caspase-3/7 activation. Both the intrinsic and extrinsic apoptosis pathways were engaged, as shown by mitochondrial depolarisation along with caspase-9 and caspase-8 activation. The compounds also increased reactive oxygen species levels, further promoting apoptosis. Notably, MM118 and MM119 triggered pyroptosis - an inflammatory form of cell death - indicated by caspase-1 activation and NF-κB translocation. In a zebrafish xenograft model, both compounds significantly reduced tumour growth. These findings highlight MM118 and MM119 as promising candidates for GBM therapy that may overcome resistance by engaging multiple cell-death pathways.

## Introduction

Glioblastoma multiforme (GBM) is the most aggressive and common primary brain tumour in adults. It is characterised by rapid growth, invasiveness, and high resistance to standard therapies such as surgery, radiotherapy, and chemotherapy^1^. The tumour is heterogeneous and can evade programmed cell death mechanisms, thus limiting the efficacy of available treatments^2^. The average survival time for patients is approximately 12–15 months from diagnosis^3^. Developing new therapeutic strategies, including compounds capable of inducing alternative forms of cell death, such as pyroptosis, is therefore essential to improve the prognosis of patients with GBM^4^^,^^5^.

Pyroptosis, unlike classical apoptosis, is an inflammatory form of programmed cell death that plays a crucial role in the innate immune response and has emerged as a potential therapeutic target in cancer treatment^6^. Unlike apoptosis, which is a relatively silent process that does not provoke an immune response, pyroptosis is characterised by intense inflammation^7^. This renders pyroptosis a particularly salient phenomenon in the context of GBM, a highly aggressive brain tumour that frequently develops resistance to conventional therapies by evading apoptosis.

The pyroptotic process is initiated by activating the inflammasome, a multiprotein complex that senses cellular stress, DNA damage, and pathogen-associated molecular patterns (PAMPs). Following inflammasome activation, the process is further facilitated by the activation of the enzyme caspase-1, which plays a pivotal role in cleaving and activating proinflammatory cytokines, as well as in promoting the formation of membrane pores. These pores result in cell swelling, plasma membrane rupture, and subsequent cell lysis, leading to the release of inflammatory mediators into the surrounding environment^8^.

Inducing pyroptosis in cancer cells is a promising alternative strategy to bypassing apoptosis resistance, a well-documented challenge in GBM and other aggressive tumours^9^. By stimulating an inflammatory form of cell death, pyroptosis not only directly eliminates tumour cells but also enhances antitumor immune responses, potentially making tumours more susceptible to immune surveillance^10^.

Triazine derivatives have emerged as a promising class of compounds with potential anticancer properties, demonstrating antiproliferative and cytotoxic effects on cancer cells^11^. These compounds have been shown to interfere with key cellular processes, such as DNA replication, cell cycle progression, and apoptosis, making them attractive candidates for further investigation in drug development. A prominent example of a triazine-based chemotherapeutic agent is temozolomide (TMZ), which is currently employed as the standard of care for the treatment of GBM. TMZ exerts its cytotoxic effects by inducing DNA damage and subsequent apoptosis^12^. The structural and functional significance of the triazine core in anticancer therapy underscores the potential of newly designed triazine derivatives as alternative therapeutic agents.

The present study focuses on two newly synthesised triazine-based compounds, MM118 and MM119, investigating their effects on glioblastoma cell lines A172 and U-87 MG. The primary objective is to assess their impact on cell viability and mechanisms of cell death, with a particular emphasis on their ability to induce pyroptosis. The study was motivated by the recognition of the critical role played by alternative cell death pathways in overcoming apoptosis resistance, a common feature of glioblastoma.

## Results

### Synthesis

**Figure 1.**
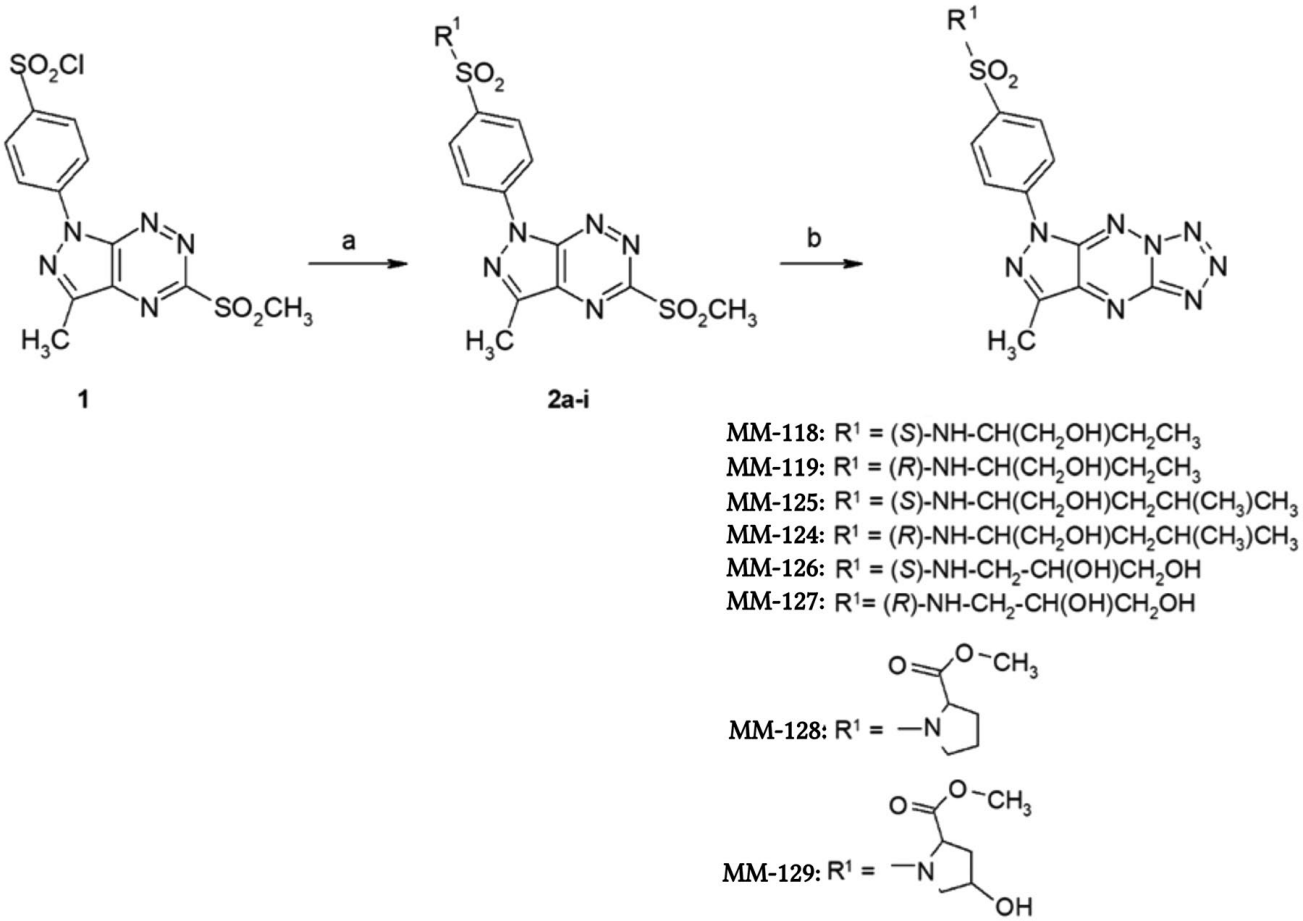
Reaction conditions: (a) reaction of derivative 1 with NH**_3_** (25% aq., 2 drops) in anhydrous MeCN at room temperature (2a), or with the appropriate amine in MeCN at rt overnight (2b–i); (b) substitution with NaN**_3_** in anhydrous ethanol under reflux.

The preparation of the final tricyclic sulphonamide derivatives was accomplished through a multistep synthetic pathway, as previously detailed in our earlier publications^13–16^. The synthesis commenced with the preparation of the key chlorosulphone derivative 1, which reacted efficiently with amines to afford the corresponding pyrazolotriazine sulphonamides 2a-i in good yields. In the following step, nucleophilic substitution of the methylsulphone group in compounds 2a-i with sodium azide in anhydrous ethanol generated the respective azide intermediates. These subsequently underwent intramolecular cyclisation under the reaction conditions to furnish the final tetrazole derivatives 3a-i (Figure 1). The structures of the newly obtained compounds were confirmed by spectroscopic analyses and molecular weight measurements.

#### Synthesis of sulphonamides (2a-i)

Derivative 1 (194 mg, 0.5 mmol) was dissolved in 5 ml of anhydrous acetonitrile and 2 drops of a 25% ammonia solution or the appropriate amine derivative (pure enantiomer, 1.75 mmol) were added. After stirring overnight at room temperature, the solvent was evaporated under reduced pressure. The crude product was purified by column chromatography on silica using a mixture of CH**_2_**Cl**_2_**:EtOH (25:1) as the eluent, yielding the target compounds. The starting compound **1** was prepared according to the method described in the literature^15^^,^^16^. For the synthesis **2i** L-Proline methyl ester hydrochloride was used and cis-4-hydroxy-D-proline methyl ester for construction **2h**.

**Synthesis of 4–(3-methyl-5-methylsulfonyl-1*H*-pyrazolo[4,3-*e*]**^1^^,^^2^^,^^4^**triazyn-1-yl)benzenesulfon-amide (2a):** Yield 85%. Melting point: 143–**145 °C**; ^1^H NMR (DMSO) δ: 2.79 (s, 3H, CH_3_), 3.62 (*s*, 3H, SO_2_CH_3_), 7.20 (bs, 1H, exchanged with D_2_O, NH), 7.50 (bs, 1H, exchanged with D_2_O, NH), 8.12 (d, 2H, *J* = 8.8 Hz), 8.52 (d, 2H, *J* = 8.8 Hz); ^13^C NMR (DMSO) δ: 11.10, 40.81, 120.02, 127.59, 138.11, 139.88, 142.27, 145.95, 148.25, 160.94; HRMS (ESI, m/z) Calcd for C_12_H_13_N_6_O_4_S_2_ [M^+^+H] 369.04342. Found [M^+^+H] 369.04388. Anal. Calcd C_12_H_12_N_6_O_4_S_2_: C, 39.12; H, 3.28; N, 22.81. Found: C, 39.32; H, 4.50; N, 22.70.

**Synthesis of *N*-(*S*)-(1-hydroxy-but-2-yl)-4–(3-methyl-5-methylsulfonyl-1*H*-pyrazolo[4,3-*e*]**^1^^,^^2^^,^^4^**triazyn-1-yl)benzenesulfonamide (2b):** Yield 80%. Melting point: 133–**135 °C**; ^1^H NMR (DMSO) δ: 0.65 (t, 3H, *J* = 7.2 Hz), 1.20–1.26 (*m*, 1H), 1.52–1.58 (*m*, 1H), 2.79 (*s*, 3H), 3.00–3.05 (*m*, 1H), 3.14–3.20 (*m*, 1H), 3.27–3.32 (*m*, 1H), 3.62 (*s*, 3H, SO_2_CH_3_), 4.66 (*t*, 1H, *J* = 5.6 Hz, exchanged with D_2_O, OH), 7.64 (d, 1H, *J* = 7.6 Hz, exchanged with D_2_O, NH), 8.10 (d, 2H, *J* = 8.8 Hz), 8.53 (d, 2H, *J* = 8.8 Hz); ^13^C NMR (DMSO) δ: 9.95, 11.10, 23.92, 40.81, 56.86, 63.29, 119.93, 128.37, 138.22, 140.11, 140.15, 146.05, 148.29, 160.96; HRMS (ESI, m/z) Calcd for C_16_H_21_N_6_O_5_S_2_ [M^+^+H] 441.10094. Found [M^+^+H] 441.10141. Anal. Calcd for C_16_H_20_N_6_O_5_S_2_: C, 43.63; H, 4.58; N, 19.08. Found: C, 43.82; H, 4.65; N, 18.87.

**Synthesis of *N*-(*R*)-(1-hydroxy-but-2-yl)-4–(3-methyl-5-methylsulfonyl-1*H*-pyrazolo[4,3-*e*]**^1^^,^^2^^,^^4^**triazyn-1-yl)benzenesulfonamide (2c):** Yield 93%. Melting point: 147–**153 °C**; ^1^H NMR (DMSO) δ: 0.65 (*t*, 3H, *J* = 7.2 Hz), 1.19–1.28 (*m*, 1H), 1.50–1.58 (*m*, 1H), 2.79 (*s*, 3H), 3.00–3.07 (*m*, 1H), 3.14–3.20 (*m*, 1H), 3.27–3.31 (*m*, 1H), 3.62 (*s*, 3H, SO_2_CH_3_), 4.65 (*t*, 1H, *J* = 5.6 Hz, exchanged with D_2_O, OH), 7.64 (d, 1H, *J* = 8.0 Hz, exchanged with D_2_O, NH), 8.09 (d, 2H, *J* = 8.8 Hz), 8.53 (d, 2H, *J* = 8.8 Hz); ^13^C NMR (DMSO) δ: 9.94, 11.09, 23.90, 40.79, 56.84, 63.27, 119.91, 128.35, 138.21, 140.10, 140.14, 146.02, 148.28, 160.94; HRMS (ESI, *m*/*z*) Calcd for C_16_H_21_N_6_O_5_S_2_ [M^+^+H] 441.10094. Found [M^+^+H] 441.1013. Anal. Calcd for C_16_H_20_N_6_O_5_S_2_: C, 43.63; H, 4.58; N, 19.08. Found: C, 43.85; H, 4.60; N, 18.92.

**Synthesis of *N*-(*S*)-(1-hydroxy-4-methyl-pent-2-yl)-4–(3-methyl-5-methylsulfonyl-1*H*-pyrazolo[4,3-*e*]**^1^^,^^2^^,^^4^**triazyn-1-yl)benzenesulfonamide (2d):** Yield 82%. Melting point: 141–**145 °C**; ^1^H NMR (DMSO) δ: 0.59 (d, 3H, *J* = 6.4 Hz), 0,76 (d, 3H, *J* = 6.4 Hz), 1.10–1.19 (*m*, 1H), 1.28–1.35 (*m*, 1H). 1.45–1.55 (*m*, 1H), 2.79 (*s*, 3H), 3.10–3.17 (*m*, 2H), 3.27 (*m*, 1H), 3.62 (*s*, 3H), 4.66 (*t*, 1H, *J* = 5.6 Hz, exchanged with D_2_O, OH), 7.65 (d, 1H, *J* = 7.2 Hz, exchanged with D_2_O, NH), 8.10 (d, 2H, *J* = 8.8 Hz), 8.53 (d, 2H, *J* = 8.8 Hz); ^13^C NMR (DMSO) δ: 11.83, 22.22, 23.99, 24.57, 41.13, 41.59, 54.13, 64.78, 121.27, 129.14, 138.90, 140.64, 141.03, 147.37, 148.89, 161.50; HRMS (ESI, *m*/*z*) Calcd for C_18_H_24_N_6_O_5_S_2_ [M^+^+H] 469.13224. Found [M^+^+H] 469.13286. Anal. Calcd for C_18_H_24_N_6_O_5_S_2_: C, 46.14; H, 5.16; N, 17.94. Found: C, 46.30; H, 5.28; N, 17.78.

**Synthesis of *N*-(*S*)-(2,3-dihydroxy-prop-1-yl)-4–(3-methyl-5-methylsulfonyl-1*H*-pyrazolo[4,3-*e*]**^1^^,^^2^^,^^4^**triazyn-1-yl)benzenesulfonamide (2f):** Yield 83%. Melting point: 137–**141 °C**; ^1^H NMR (DMSO) δ: 2.65–2.72 (*m*, 1H), 2.79 (*s*, 3H), 2.92–2.98 (*m*, 1H), 3.25–3.29 (*m*, 2H), 3.46–3.51 (*m*, 1H), 3.62 (*s*, 3H, SO_2_CH_3_), 4.56 (*t*, 1H, *J* = 5.6 Hz, exchanged with D_2_O, OH), 4.80 (d, 2H, *J* = 5.2 Hz, exchanged with D_2_O, OH), 7.67 (*t*, 1H, *J* = 6.4 Hz, exchanged with D_2_O, NH), 8.09 (d, 2H, *J* = 8.8 Hz), 8.54 (d, 2H, *J* = 8.8 Hz); ^13^C NMR (DMSO) δ: 11.12, 40.80, 46.10, 63.47, 70.36, 120.10, 128.55, 138.20, 138.63, 140.28, 146.05, 148.31, 160.98; HRMS (ESI, *m*/*z*) Calcd for C_15_H_19_N_6_O_6_S_2_ [M^+^+H] 443.08020. Found [M^+^+H] 443.08058. Anal. Calcd for C_15_H_18_N_6_O_6_S_2_: C, 40.72; H, 4.10; N, 18.99. Found: C, 40.58; H, 4.28; N, 18.74.

**Synthesis of *N*-(*R*)-(2,3-dihydroxy-prop-1-yl)-4–(3-methyl-5-methylsulfonyl-1*H*-pyrazolo[4,3-*e*]**^1^^,^^2^^,^^4^**triazyn-1-yl)benzenesulfonamide (2 g):** Yield 84%. Melting point: 139–**142 °C**; ^1^H NMR (DMSO) δ: 2.65–2.70 (*m*, 1H), 2.79 (*s*, 3H), 2.93–2.98 (*m*, 1H), 3.25–3.30 (*m*, 2H), 3.44–3.50 (*m*, 1H), 3.63 (*s*, 3H, SO_2_CH_3_), 4.55 (*t*, 1H, *J* = 5.6 Hz, exchanged with D_2_O, OH), 4.79 (d, 2H, *J* = 4.8 Hz, exchanged with D_2_O, OH), 7.68 (*t*, 1H, *J* = 6.4 Hz, exchanged with D_2_O, NH), 8.09 (d, 2H, *J* = 8.8 Hz), 8.54 (d, 2H, *J* = 8.8 Hz); ^13^C NMR (DMSO) δ: 11.55, 41.28, 46.26, 63.67, 70.62, 120.85, 128.97, 138.61, 138.81, 140.85, 146.80, 148.66, 161.31; HRMS (ESI, *m*/*z*) Calcd for C_15_H_19_N_6_O_6_S_2_ [M^+^+H] 443.08020. Found [M^+^+H] 443.08046. Anal. Calcd for C_15_H_18_N_6_O_6_S_2_: C, 40.72; H, 4.10; N, 18.99. Found: C, 40.68; H, 4.15; N, 18.92.

**Synthesis of methyl 1-[4–(3-methyl-5-methylsulfonyl-1*H*-pyrazolo[4,3-*e*]**^1^^,^^2^^,^^4^**triazin-1-yl)phenyl-sulfonyl]pyrrolidine-2-carboxylate (2h):** Yield 89%. Melting point: 79–**83 °C**; ^1^H NMR (DMSO) δ: ^1^H NMR (DMSO) δ: 1.64–1.68 (*m*, 1H), 1.80–2.03 (*m*, 3H), 2.79 (*s*, 3H), 3.23–3.28 (*m*, 1H), 3.42–3.48 (*m*, 1H), 3.63 (*s*, 3H), 3.67 (*s*, 3H), 4.32 (dd, 1H, J_1_ = 9.2 Hz, J_2_ = 4.4 Hz), 8.15 (d, 2H, *J* = 9.2 Hz), 8.59 (d, 2H, *J* = 9.2 Hz); ^13^C NMR (CDCl_3_) δ: 11.12, 24.28, 30.45, 40.78, 48.54, 52.23, 60.33, 120.15, 129.22, 135.24, 138.40, 140.97, 146.27, 148.41, 161.04, 172.08. HRMS (ESI, *m*/*z*) Calcd for C_18_H_21_N_6_O_6_S_2_ [M^+^+H] 481.09585. Found [M^+^+H] 481.09645. Anal. Calcd for C_18_H_20_N_6_O_6_S_2_: C, 44.99; H, 4.20; N, 17.49. Found: C, 44.73; H, 4.23; N, 17.39.

**(*Z*)-4-hydroxy-1-[4–(3-methyl-5-methylsulfonyl-1*H*-pyrazolo[4,3-*e*]**^1^^,^^2^^,^^4^**triazin-1-yl)phenylsul-fonyl]pyrrolidine-2-carboxylate (2i):** Yield 96%. Melting point: 112–**114 °C**; ^1^H NMR (acetone) δ: 2.07–2.13 (*m*, 1H), 2.15–2.21 (*m*, 1H), 2.85 (*s*, 3H), 3.45 (dt, 1H, J_1_ = 11.2 Hz, J_2_ = 1.7 Hz), 3.59 (*s*, 3H), 3.65 (dd, 1H, J_1_ = 10.8 Hz, J_2_ = 4.2 Hz), 3.73 (*s*, 3H), 4.36 (*t*, 1H, *J* = 7.9 Hz), 4.42 (bs, 1H), 8.16 (d, 2H, *J* = 8.7 Hz), 8.67 (d, 2H, *J* = 9.1 Hz); ^13^C NMR (acetone) δ: 11.26, 23.33, 40.21, 41.15, 57.60, 60.83, 70.11, 120.80, 130.32, 137.24, 142.19, 147.31, 149.60, 162.92, 173.10. HRMS (ESI, *m*/*z*) Calcd for C_18_H_20_N_6_O_7_S_2_ [M^+^+H] 497.0908 Found [M^+^+H] 497.0904. Anal. Calcd for C_18_H_20_N_6_O_7_S_2_: C, 43.54; H, 4.06; N, 16.93. Found: C, 43.60; H, 4.15; N, 16.78.

#### Synthesis of tricyclic sulphonamides (3ab)

Sulphonamide derivative bearing a methylsulphonyl group (0.33 mmol) was dissolved in anhydrous ethanol (5 ml), followed by the addition of sodium azide (26 mg, 0.40 mmol). The reaction mixture was refluxed until complete consumption of the starting material, as monitored by TLC. Subsequently, the solvent was evaporated under reduced pressure, and the crude product was purified by column chromatography using CH_2_Cl_2_:MeOH (50:1) as the eluent, affording the target compounds as an orange-red solid^15^^,^^16^.

**4-[7-methyl-*5H*-pyrazolo[4,3-*e*]tetrazolo[1,5-*b*]**^1^^,^^2^^,^^4^**triazin-5-yl)]benzenesulfonamide (3a)**: Yield 78%. Melting point: 165–167 °C; ^1^H NMR (DMSO) δ: 3.57 (*s*, 3H), 8.90 (d, 2H, *J* = 8.4 Hz), 9.11 (d, 2H, *J* = 8.4 Hz); ^13^C NMR (DMSO) δ: 11.15, 120.08, 127.49, 138.11, 139.80, 142.22, 145.95, 148.25, 160.90; HRMS (ESI, *m*/*z*) Calcd for C_15_H_17_N_9_O_3_S [M^+^+H] 330.05162. Found [M^+^+H] 330.05262. Anal. Calcd for C_11_H_9_N_9_O_2_S: C, 39.88,; H, 2.74; N, 38.05. Found: C, 39.78; H, 2.80; N, 37.92.

***N*-(*R*)-(1-hydroxy-but-2-yl)-4-[7-methyl-*5H*-pyrazolo[4,3-*e*]tetrazolo[1,5-*b*]**^1^^,^^2^^,^^4^**triazin-5-yl)]benzenesulfonamide (3b: MM-118):** Yield 58%. Melting point: 175–177 °C; ^1^H NMR (methanol) δ: 0.74 (*t*, 3H, J = 7.6 Hz), 1.28–1.36 (*m*, 1H), 1.58–1.63 (*m*, 1H), 2.85 (*s*, 3H), 3.11–3.18 (*m*, 1H), 3.34–3.38 (*m*, 1H), 3.44–3.48 (*m*, 1H), 4.57 (bs, 1H, OH), 8.10 (d, 2H, *J* = 8.8 Hz), 8.44 (d, 2H, *J* = 8.8 Hz); ^13^C NMR (methanol) δ: 10.50, 11.17, 25.49, 58.43, 65.18, 120.13, 129.68, 140.88, 141.88, 143.33, 148.29, 148.82, 149.32; HRMS (ESI, *m*/*z*) Calcd for C_15_H_17_N_9_O_3_S [M^+^+H] 404.12478. Found [M^+^+H] 404.12431. Anal. Calcd for C_15_H_17_N_9_O_3_S: C, 44.66; H, 4.25; N, 31.25. Found: C, 44.80; H, 4.40; N, 31.10.

***N*-(*S*)-(1-hydroxy-but-2-yl)-4-[7-methyl-5*H*-pyrazolo[4,3-*e*]tetrazolo[1,5-*b*]**^1^^,^^2^^,^^4^**triazin-5-yl)]benzenesulfonamide (3c: MM-119)**: Yield 86%. Melting point: 174–176 °C; ^1^H NMR (methanol) δ: 0.74 (*t*, 3H, *J* = 7.6 Hz), 1.28–1.36 (*m*, 1H), 1.58–1.65 (*m*, 1H), 2.85 (*s*, 3H), 3.11–3.19 (*m*, 1H), 3.34–3.38 (*m*, 1H), 3.44–3.48 (*m*, 1H), 4.57 (bs, 1H, OH), 8.10 (d, 2H, *J* = 8.8 Hz), 8.44 (d, 2H, *J* = 8.8 Hz); ^13^C NMR (methanol) δ: 10.50, 11.17, 25.49, 58.43, 65.18, 120.12, 129.68, 140.88, 141.88, 143.32, 148.29, 148.83, 149.32; HRMS (ESI, m/z) Calcd for C_15_H_17_N_9_O_3_S [M^+^+H] 404.12478. Found [M^+^+H] 404.12485. Anal. Calcd for C_15_H_17_N_9_O_3_S: C, 44.66; H, 4.25; N, 31.25. Found: C, 44.78; H, 4.48; N, 31.04.

***N*-(*S*)-(1-hydroxy-4-methyl-pent-2-yl)-4-[7-methyl-5*H*-pyrazolo[4,3-*e*]tetrazolo[1,5-*b*]**^1^^,^^2^^,^^4^**triazin-5-yl)]benzenesulfonamide (3d: MM-125): Yield 88%.** Melting point: 159–**161 °C**; ^1^H NMR (methanol) δ: 0.66 (d, 3H, *J* = 6.4 Hz), 0,81 (d, 3H, *J* = 6.4 Hz), 1.20–1.26 (*m*, 1H), 1.32–1.38 (*m*, 1H), 1.48–1.55 (*m*, 1H), 2.85 (*s*, 3H), 3.25–3.35 (*m*, 2H), 3.42–3.46 (*m*, 1H), 4.57 (bs, 1H, OH), 8.08 (d, 2H, *J* = 8.8 Hz), 8.43 (d, 2H, *J* = 8.8 Hz); ^13^C NMR (methanol) δ: 11.18, 22.05, 23.62, 25.35, 42.03, 54.89, 65.96, 120.13, 129.70, 140.91, 141.86, 143.32, 148.33, 148.87, 149.31; HRMS (ESI, *m*/*z*) Calcd for C_17_H_21_N_9_O_3_S [M^+^+H] 432,48235. Found [M^+^+H] 432,48282. Anal. Calcd for C_17_H_21_N_9_O_3_S: C, 47.32; H, 4.91; N, 29.22. Found: C, 47.55; H, 4.88; N, 29.05.

***N*-(*R*)-(1-hydroxy-4-methyl-pent-2-yl)-4-[7-methyl-*5*H-pyrazolo[4,3-*e*]tetrazolo[1,5-*b*]**^1^^,^^2^^,^^4^**triazin-5-yl)]benzenesulfonamide (3e: MM-124):** Yield 76%. Melting point: 157–**160 °C**; ^1^H NMR (methanol) δ: 0.66 (d, 3H, *J* = 6.4 Hz), 0,81 (d, 3H, *J* = 6.8 Hz), 1.18–1.26 (*m*, 1H), 1.32–1.38 (*m*, 1H), 1.48–1.55 (*m*, 1H), 2.85 (*s*, 3H), 3.24–3.30 (*m*, 1H), 3.30–3.35 (*m*, 1H), 3.42–3.45 (*m*, 1H), 4.57 (*t*, 1H, J = 5.6 Hz, OH), 8.08 (d, 2H, *J* = 8.8 Hz), 8.43 (d, 2H, *J* = 8.8 Hz); ^13^C NMR (methanol) δ: 11.19, 22.05, 23.62, 25.34, 42.02, 54.88, 65.95, 120.13, 129.68, 140.88, 141.86, 143.30, 148.33, 148.88, 149.30; HRMS (ESI, *m*/*z*) Calcd for C_17_H_21_N_9_O_3_S [M^+^+H] 432,48235. Found [M^+^+H] 432,48276. Anal. Calcd for C_17_H_21_N_9_O_3_S: C, 47.32; H, 4.91; N, 29.22. Found: C, 47.49; H, 5.08; N, 29.00.

***N*-(*S*)-(2,3-dihydroxy-prop-1-yl)-4-[7-methyl-5*H*-pyrazolo[4,3-*e*]tetrazolo[1,5-*b*]**^1^^,^^2^^,^^4^**triazin-5-yl)]benzene-sulphonamide (3f: MM-126):** Yield 73%. Melting point: 167–**171 °C**; ^1^H NMR (MeOH) δ: 2.85 (*s*, 3H), 2.86–2.90 (*m*, 1H), 3.04–3.09 (*m*, 1H), 3.47–3.50 (*m*, 2H), 3.63–3.68 (*m*, 1H), 4.58 (bs, 1H, NH), 8.09 (d, 2H, *J* = 8.8 Hz), 8.46 (d, 2H, *J* = 8.8 Hz); ^13^C NMR (DMSO) δ: 11.18, 46.87, 64.84, 72.00, 120.26, 129.77, 139.35, 142.04, 143.34, 148.30, 148.87, 149.32; HRMS (ESI, *m*/*z*) Calcd for C_14_H_14_N_9_O_4_S [M^+^+H] 404.08840. Found [M^+^+H] 404.08930. Anal. Calcd for C_14_H_15_N_9_O_4_S: C, 41.48; H, 3.73; N, 31.10. Found: C, 41.25; H, 3.88; N, 29.92.

***N*-(*R*)-(2,3-dihydroxy-prop-1-yl)-4-[7-methyl-5*H*-pyrazolo[4,3-*e*]tetrazolo[1,5-*b*]**^1^^,^^2^^,^^4^**triazin-5-yl)]benzene-sulphonamide (3 g: MM-127):** Yield 73%. Melting point: 169–**172 °C**; ^1^H NMR (MeOH) δ: 2.85 (*s*, 3H), 2.86–2.90 (*m*, 1H), 3.04–3.09 (*m*, 1H), 3.47–3.50 (*m*, 2H), 3.63–3.68 (*m*, 1H), 4.57 (bs, 1H, NH), 8.09 (d, 2H, *J* = 8.8 Hz), 8.46 (d, 2H, *J* = 8.8 Hz); ^13^C NMR (DMSO) δ: 11.18, 46.87, 64.84, 72.00, 120.26, 129.77, 139.34, 142.04, 143.33, 148.30, 148.88, 149.32; HRMS (ESI, *m*/*z*) Calcd for C_14_H_14_N_9_O_4_S [M^+^+H] 404.08840. Found [M^+^+H] 404.08931. Anal. Calcd for C_14_H_15_N_9_O_4_S: C, 41.48; H, 3.73; N, 31.10. Found: C, 41.30; H, 3.90; N, 29.88.

**Methyl 1-[4–(7-methyl-5*H*-pyrazolo[4,3-*e*]tetrazolo[1,5-*b*]**^1^^,^^2^^,^^4^**triazin-5-yl)phenylsulfonyl]-pyrrolidine-2-carboxylate (3h: MM-128):** Yield 93%. Melting point: 125–**127 °C**; ^1^H NMR (DMSO) δ: 1.62–1.68 (*m*, 1H), 1.80–1.95 (*m*, 2H), 1.96–2.01 (*m*, 1H), 2.79 (*s*, 3H), 3.22–3.27 (*m*, 1H), 3.38–3.45 (*m*, 2H), 3.66 (*s*, 3H), 8.15 (d, 2H, *J* = 8,8 Hz), 8.40 (d, 2H, *J* = 8,8 Hz); ^13^C NMR (DMSO) δ: 11.11, 24.31, 30.48, 48.59, 52.27, 60.38, 119.08, 129.43, 134.66, 138.65, 140.58, 143.46, 147.55, 163.13, 172.14; HRMS (ESI, *m*/*z*) Calcd for C_17_H_18_N_9_O_4_S [M^+^+H] 444.11970. Found [M^+^+H] 444.11993. Anal. Calcd for C_17_H_17_N_9_O_4_S: C, 46.05; H, 3.86; N, 28.43. Found: C, 45.85; H, 3.89; N, 28.31.

**Methyl (*Z*)-4-hydroxy-1-[4–(7-methyl-5*H*-pyrazolo[4,3-*e*]tetrazolo[1,5-*b*]**^1^^,^^2^^,^^4^**triazin-5-yl)phenyl-sulfonyl]pyrrolidine-2-carboxylate (3i: MM-129):** Yield 83%. Melting point: 122–**128 °C**; ^1^H NMR (acetone) δ: 2.04–2.18 (*m,* 2H), 3.39 (d, 1H, *J* = 11.2 Hz), 2.85 (*s*, 3H), 3.62 (d, 1H, *J* = 12 Hz), 3.77 (*s*, 3H), 4.33 (d, 2H, *J* = 8.3 Hz), 4.57 (bs, 1H, OH), 8.08 (d, 2H, *J* = 9.2 Hz), 8.48 (d, 2H, *J* = 9.2 Hz); ^13^C NMR (acetone) δ: 11.26, 41.14, 52.53, 57.59, 60.82, 70.22, 120.79, 130.32, 137.23, 142.19, 147.30, 149.60, 162.92, 173.09. HRMS (ESI, *m*/*z*) Calcd for C_17_H_17_N_9_O_5_S [M^+^+H] 459.10734. Found [M^+^+H] 460.11469. Anal. Calcd for C17H17N9O4S: C, 44.44; H, 3.73; N, 27.44. Found: C, 44.35; H, 3.89; N, 27.20.

### The MM118 and MM119 compounds demonstrated notable cytotoxicity against glioblastoma cell lines

An MTT assay was conducted to preliminarily assess the cytotoxic effects of the new sulphonamide derivatives with a triazine group, **MM118, MM119, MM124, MM125, MM126, MM127, MM128, and MM129.** This assay measures the metabolic activity of cells and allows the determination of their survival after exposure to a given compound. The study concentrated on the impact of these compounds on human glioblastoma multiforme cells (cell lines A172, U-87 MG, U118MG, T98G) and mouse astrocytes (line C8-D1A). Following a 24-h treatment period with the compounds, the test cells exhibited IC_50_ values ranging from 0.471 to above 10 μM (Table 1). **MM118** and **MM119** compounds demonstrated superior activity-to-toxicity ratios compared to mouse astrocytic cells (C8-D1A). Consequently, these compounds were selected for subsequent studies. The A172 and U-87 MG cell lines were selected for comparative analysis due to their demonstrated genetic and treatment response variations, thereby enabling the assessment of compound efficacy across different glioma subtypes.

**Table 1.** IC_50_ values ± SEM [µM] calculated from MTT assay for the tested compounds and temozolomide (TMZ) as positive control after 24 h of incubation with cells.

	A172	U-87 MG	U-118 MG	T98G	C8-D1A
**MM118**	0.889 ± 0.243	0.992 ± 0.370	0.471 ± 0.191	2.444 ± 0.393	2.186 ± 0.340
**MM119**	1.236 ± 0.386	1.193 ± 0.422	0.670 ± 0.173	4.408 ± 1.186	2.250 ± 0.294
**MM124**	>10.000	>10.000	>10.000	>10.000	>10.000
**MM125**	1,07 ± 0.312	0,65 ± 0.431	0,49 ± 0.32	0,85 ± 0.23	0,68 ± 0.451
**MM126**	>10.000	>10.000	>10.000	>10.000	>10.000
**MM127**	>10.000	>10.000	>10.000	>10.000	>10.000
**MM128**	1,651 ± 0.296	1,3 ± 0.16	0,563 ± 0.385	1,532 ± 0.253	0,66 ± 0.197
**MM129**	2,43 ± 0.341	2,352 ± 0.177	2,13 ± 0.237	2,93 ± 0.425	2,864 ± 0.311
**TMZ**	>10.000	>10.000	>10.000	>10.000	>10.000

**Figure 2.**
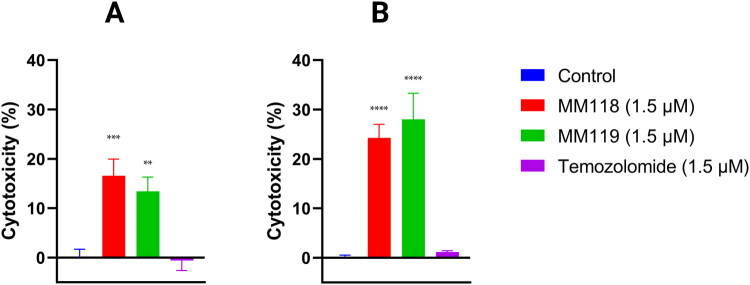
Results of LDH measurement of A172 (A) and U-87 MG (B) glioblastoma cells after 24 h of incubation with compounds. Means ± SD are shown with *N* = 3. ANOVA tests were used to demonstrate differences between cells treated with compounds and control. **P ≤ 0.01; ***P ≤ 0.001; ****P ≤ 0.0001.

In the lactate dehydrogenase (LDH) release assay, which evaluates the degree of cell membrane damage and toxicity, it was observed that compounds **MM118** and **MM119** exhibited significant toxicity against the A172 and U-87 MG cell lines. For the A172 cell line, the values were 16.59 ± 1.71% for **MM118**, 13.44 ± 2.87% for **MM119**, and −0.61 ± 2.02% for **TMZ** (1.5 μM), respectively. Conversely, for the U-87 MG line, the values were higher, reaching 24.27 ± 2.74%, 28.02 ± 5.27%, and 1.18 ± 0.25%, respectively (Figure 2). These results suggest that U-87 MG is more sensitive to **MM118** and **MM119** compared to A172. In addition, **TMZ** toxicity was significantly lower in both cell lines.

**Figure 3.**
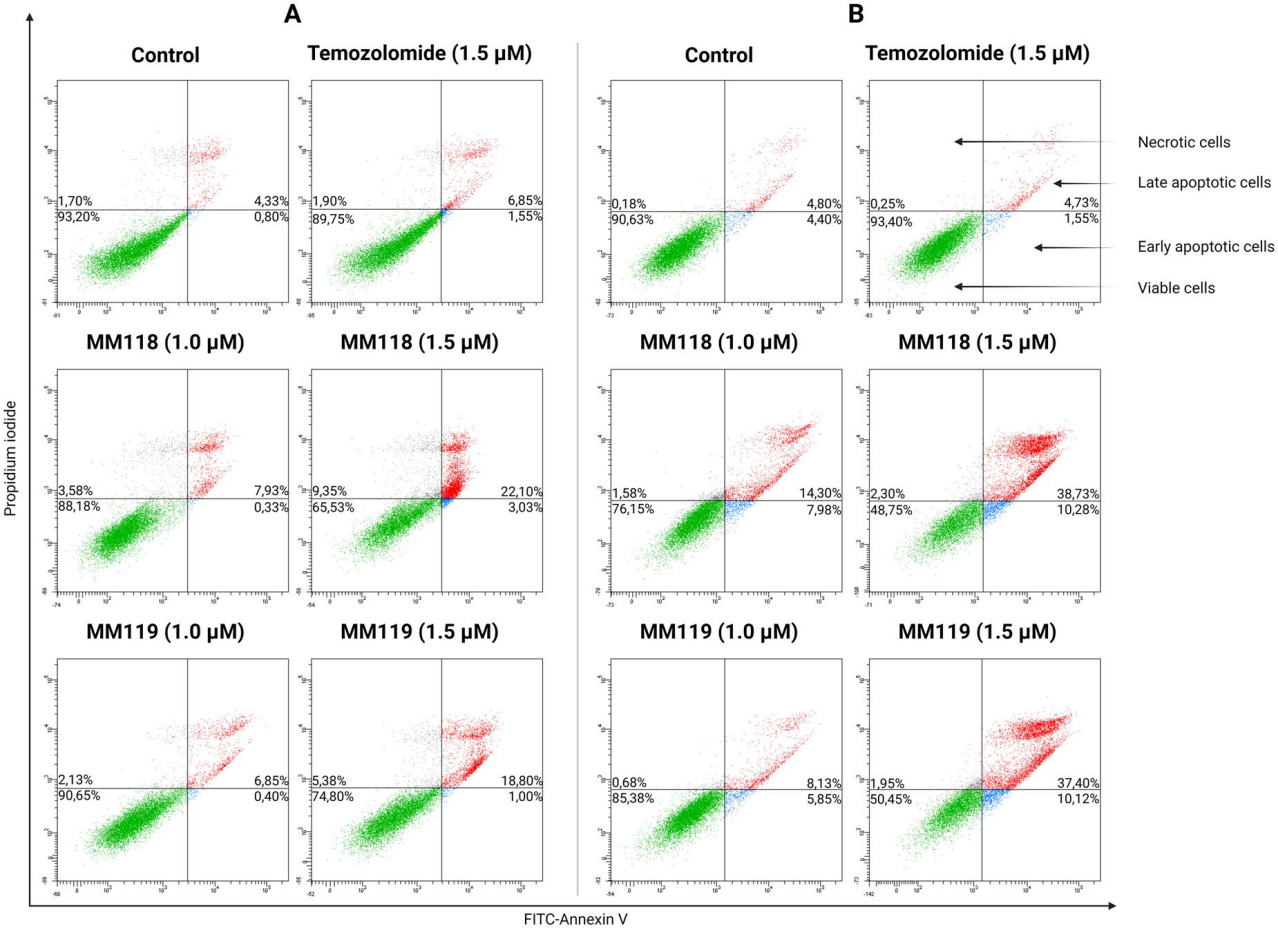
Results of flow cytometry analysis of A172 (A) and U-87 MG (B) glioblastoma cells after 24 h of incubation with compounds. Means ± SD are shown with *N* = 4.

**Figure 4.**
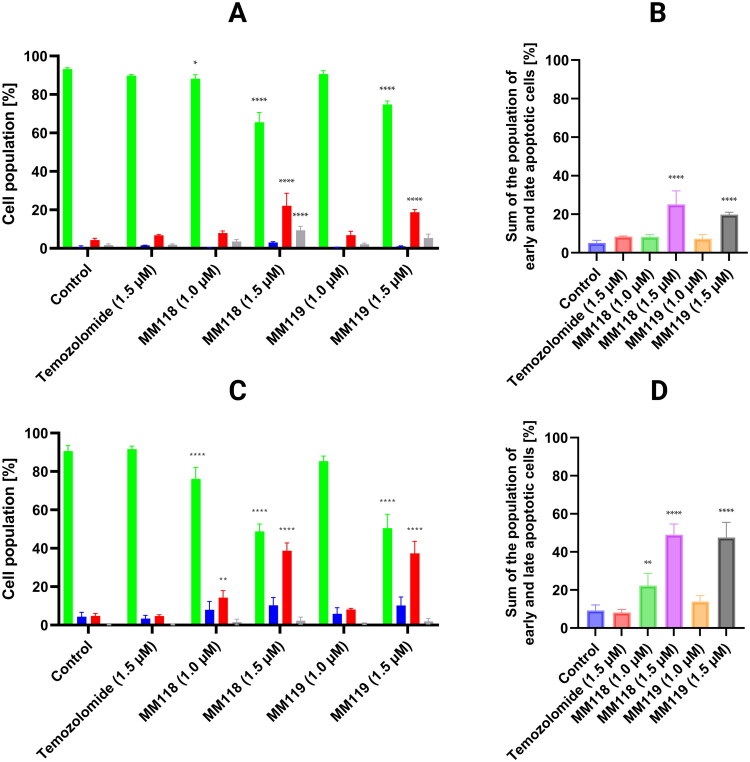
Results of flow cytometry analysis; The top graphs visualise the cytograms from the compound analysis of A172 (A) and U-87 MG (C) cells after 24 h of incubation with compounds (green bar - viable cells, blue bar - early apoptosis cells, red bar - late apoptosis cells, gray bar - necrotic cells), bottom graph shows the totals of the apoptotic cell populations for each sample of A172 (B) and U-87 MG (D) cells. Means ± SD are shown with *N* = 4. ANOVA tests were used to demonstrate differences between cells treated with compounds and control. *P ≤ 0.05; **P ≤ 0.01; ****P ≤ 0.0001.

**Figure 5.**
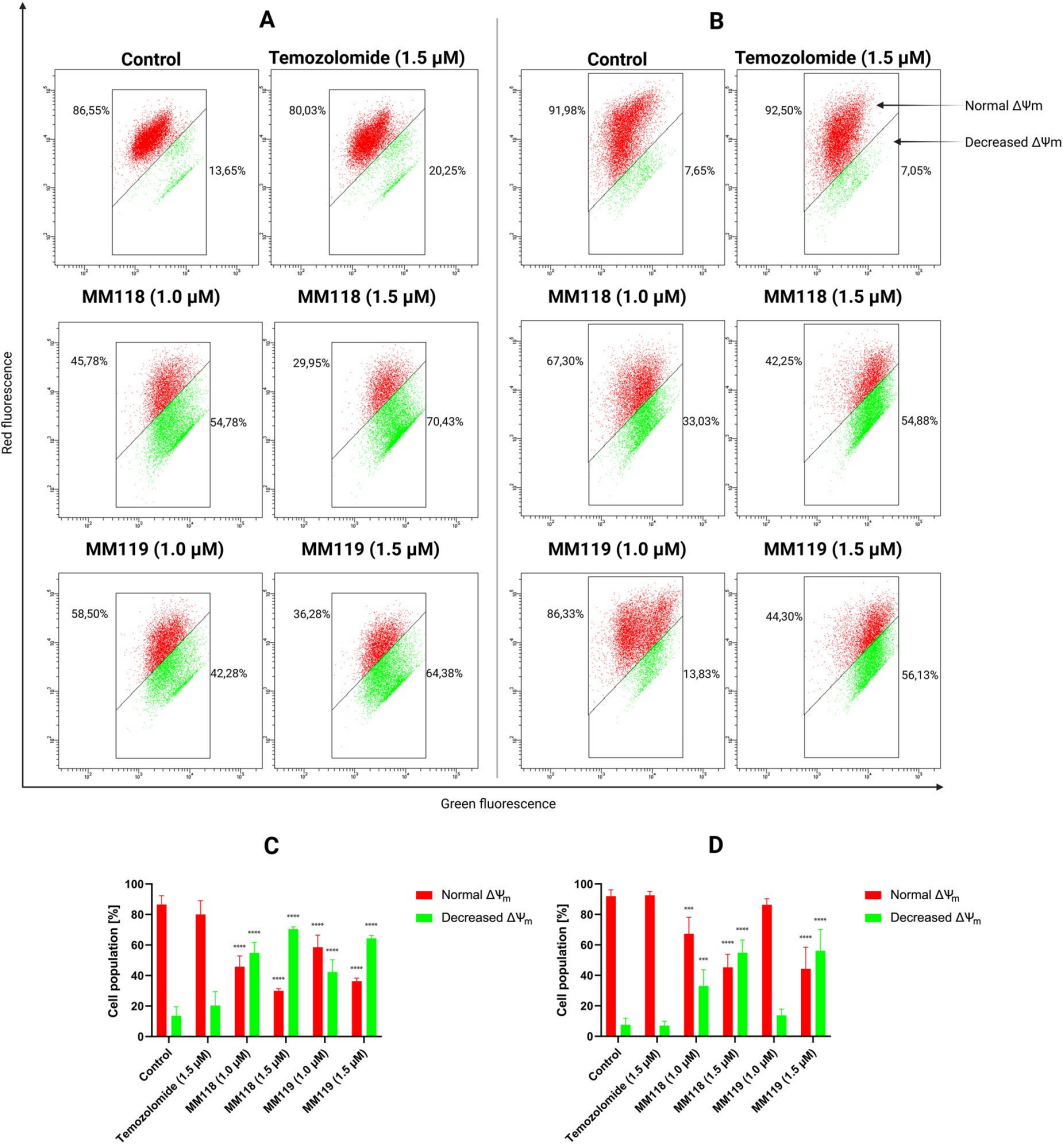
Results of flow cytometry analysis of A172 (A) and U-87 MG (B) glioblastoma cells after 24 h of incubation with compounds. Graphs visualise the cytograms from the compound analysis A172 (C) and U-87 MG (D). Means ± SD are shown with *N* = 4. ANOVA tests were used to demonstrate differences between cells treated with compounds and control. ***P ≤ 0.001; ****P ≤ 0.0001.

**Figure 6.**
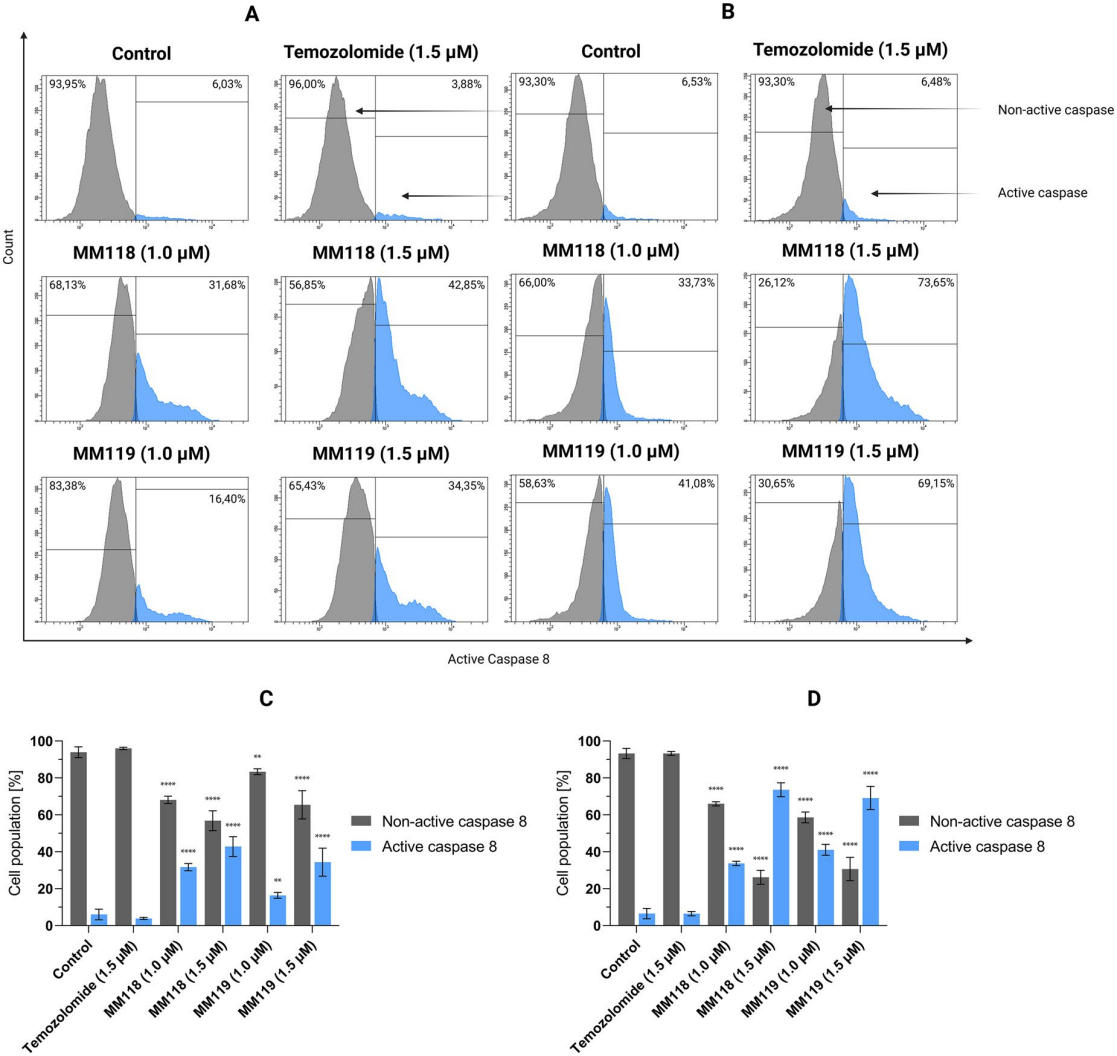
Results of flow cytometry analysis of caspase 8 activation in A172 (A) and U-87 MG (B) glioblastoma cells after 24 h of incubation with compounds. Graphs visualise the cytograms from the compound analysis A172 (C) and U-87 MG (D). Means ± SD are shown with *N* = 4. ANOVA tests were used to demonstrate differences between cells treated with compounds and control. **P ≤ 0.01; ***P ≤ 0.001; ****P ≤ 0.0001.

**Figure 7.**
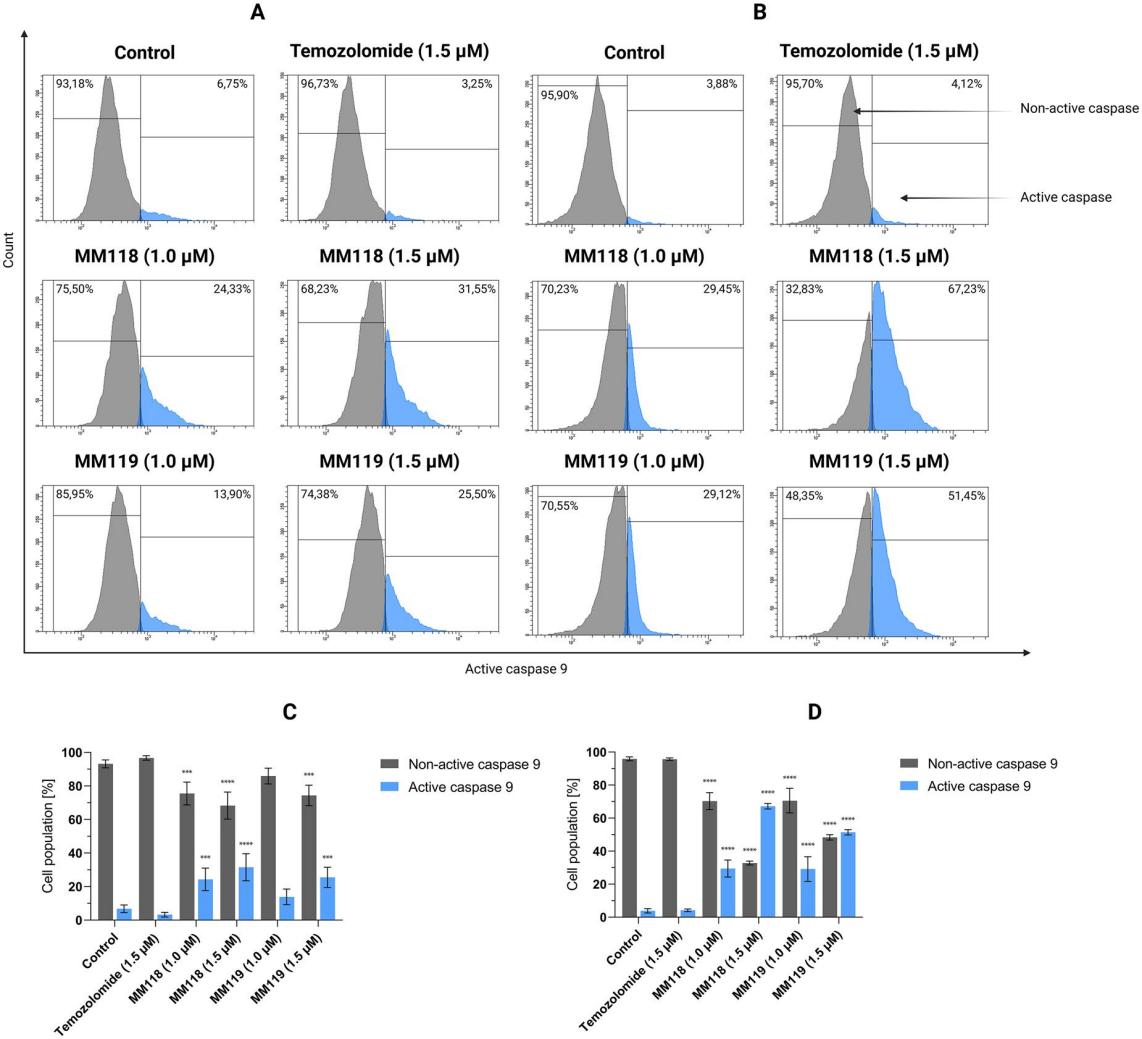
Results of flow cytometry analysis of caspase 9 activation in A172 (A) and U-87 MG (B) glioblastoma cells after 24 h of incubation with compounds. Graphs visualise the cytograms from the compound analysis A172 (C) and U-87 MG (D). Means ± SD are shown with *N* = 4. ANOVA tests were used to demonstrate differences between cells treated with compounds and control. ***P ≤ 0.001; ****P ≤ 0.0001.

**Figure 8.**
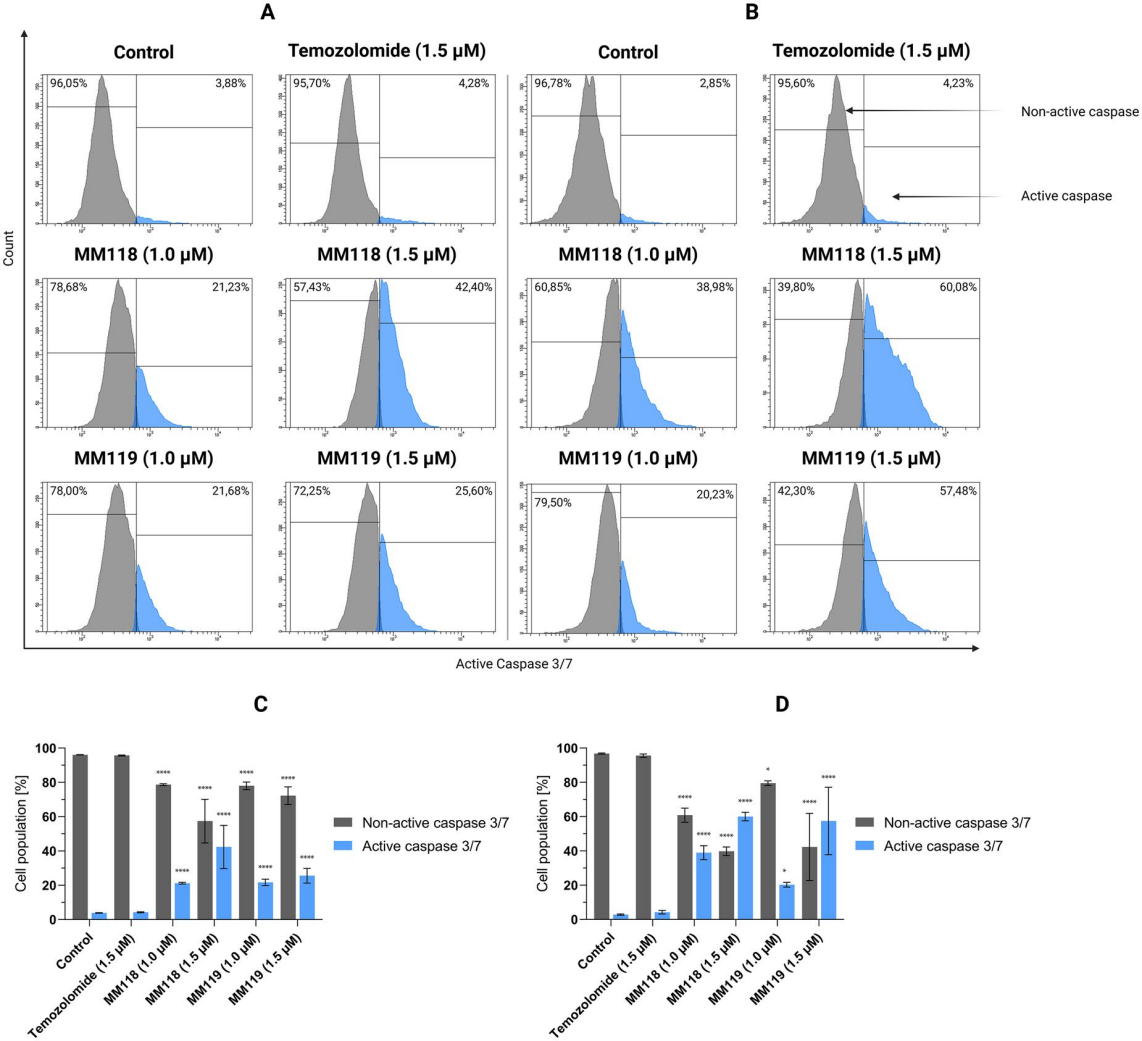
Results of flow cytometry analysis of caspase 3/7 activation in A172 (A) and U-87 MG (B) glioblastoma cells after 24 h of incubation with compounds. Graphs visualise the cytograms from the compound analysis A172 (C) and U-87 MG (D). Means ± SD are shown with *N* = 4. ANOVA tests were used to demonstrate differences between cells treated with compounds and control. *P ≤ 0.05; ***P ≤ 0.001; ****P ≤ 0.0001.

### Apoptosis-induced glioblastoma cell death by MM118 and MM119

The level of apoptosis induction of the newly synthesised compounds **MM118** and **MM119** was determined using double staining with Annexin V-FITC and propidium iodide (AV/PI). This method enables the differentiation of four cell populations based on their state: viable cells, necrotic cells, cells in early apoptosis, and late apoptosis. The compounds were analysed on A172 and U-87 MG human glioblastoma multiforme cell lines using concentrations of 1 μM and 1.5 μM over a 24-h exposure period.

The A172 cell line for **MM118** (1.5 μM) exhibited a population of viable cells at 65.53 ± 5.06%, with early apoptosis at 3.03 ± 0.44%, late apoptosis at 22.10 ± 6.56%, and necrotic cells at 9.50 ± 2.10%. For **MM119** (1.5 μM), the respective percentages were as follows: 74.80 ± 1.84%, 1.00 ± 0.28%, 18.80 ± 1.35% and 2.13 ± 0.62%. And for **TMZ** (1.5 μM), the values were as follows: 89.75 ± 0.71%, 1.55 ± 0.06%, 6.83 ± 0.39% and 1.90 ± 0.38% (Figures 3(A), 4(A,B)).

The following results were observed for the U-87 MG cell line: for **MM118** (1.5 μM), 48.75 ± 3.90% viable cells, 10.28 ± 4.08% early apoptosis, 38.73 ± 4.09% late apoptosis and 2.30 ± 1.81% necrotic cells. For **MM119** (1.5 μM), the corresponding values were as follows: 50.45 ± 7.23%, 10.18 ± 4.45%, 37.40 ± 6.20% and 1.95 ± 1.42%. The percentages for **TMZ** (1.5 μM) were: 91.65 ± 1.58%, 3.40 ± 1.63%, 4.73 ± 0.64%, and 0.25 ± 0.24% (Figures 3(B), 4(C,D)).

The impact of the novel compounds on mitochondrial membrane potential (ΔΨ_m_) was examined. Alterations in membrane potential may suggest the initiation of apoptosis via an intrinsic pathway. In this study, the fluorochrome JC-1 was employed, which, in cells with normally functioning mitochondria, aggregates and accumulates in the hyperpolarized mitochondrial membrane, emitting a red colour. Conversely, when the membrane is damaged, JC-1 breaks down into monomers, emitting green fluorescence. Both **MM118** (1.5 μM) and **MM119** (1.5 μM) have been observed to induce a decrease in mitochondrial membrane potential, with a mean value of 70.43 ± 1.44% and 64.38 ± 1.90%, respectively on A172 cell line. For the U-87 MG cell line, these parameters were equal to 54.88 ± 8.36% and 56.13 ± 13.97% of the cell population. The administration of **TMZ** (1.5 μM) resulted in a reduction of ΔΨ_m_ by 20.25 ± 9.20% in the A172 cell population and 7.05 ± 2.85% in the U-87 MG cell population (Figure 5). These findings suggest the involvement of the tested compounds in the intrinsic mitochondrial-mediated apoptosis pathway.

To corroborate our prior findings on apoptosis, we undertook an investigation into the impact of compounds on caspases activity. Caspases are proteolytic enzymes that play a pivotal role in programmed cell death, or apoptosis. Caspase 8 is activated by extracellular signals and plays a pivotal role in the initiation of apoptosis, frequently in response to cellular damage or environmental stress. Caspase 9 is involved in the intrinsic pathway, via a signal from cytochrome c, and its activation leads to the activation of apoptotic mechanisms in response to DNA damage. In contrast, caspases 3 and 7 act as effectors, leading to the activation of the apoptotic process through the degradation of proteins essential for maintaining cell integrity.

**MM118** (1.5 μM) and **MM119** (1.5 μM) cause caspase 8 activation for both cell lines in 42.85 ± 5.36% and 34.35 ± 7.58% of the A172 cell population and 73.65 ± 3.76% and 69.15 ± 6.30% of the U-87 MG cell population, respectively (Figure 6). No significant statistical differences were observed for TMZ compared to the control. These findings substantiate the role of the extrinsic pathway in the cell death process.

In contrast, a subsequent study revealed a notable elevation in the proportion of cells exhibiting active caspase 9. In A172 cells, the percentage of active caspase was 31.55 ± 8.07% for **MM118** (1.5 μM) and 25.50 ± 6.04% for **MM119**. While in the U-87 MG line it was 67.23 ± 1.66% and 51.45 ± 1.63%, respectively. As with caspase 8, no significant differences were observed between the treatment and control groups in response to **TMZ** (Figure 7). This confirms the effect of the compounds on the intrinsic apoptosis pathway.

As a concluding measure, the level of cells exhibiting activated caspase 3/7, an effector caspase, was evaluated. At a concentration of 1.5 μM, both compounds demonstrated robust activation of this caspase in both cell lines. For the A172 cell line, the level of activation was 42.40 ± 12.59% for **MM118** and 25.60 ± 4.29% for **MM119**. For U-87 MG, the activation was 60.08 ± 2.50% for **MM118** and 57.48 ± 19.68% for **MM119**. As with the preceding analyses, no significant differences were observed for TMZ (1.5 μM) (Figure 8).

### MM118 and MM119 cause the activation of reactive oxygen species

**Figure 9.**
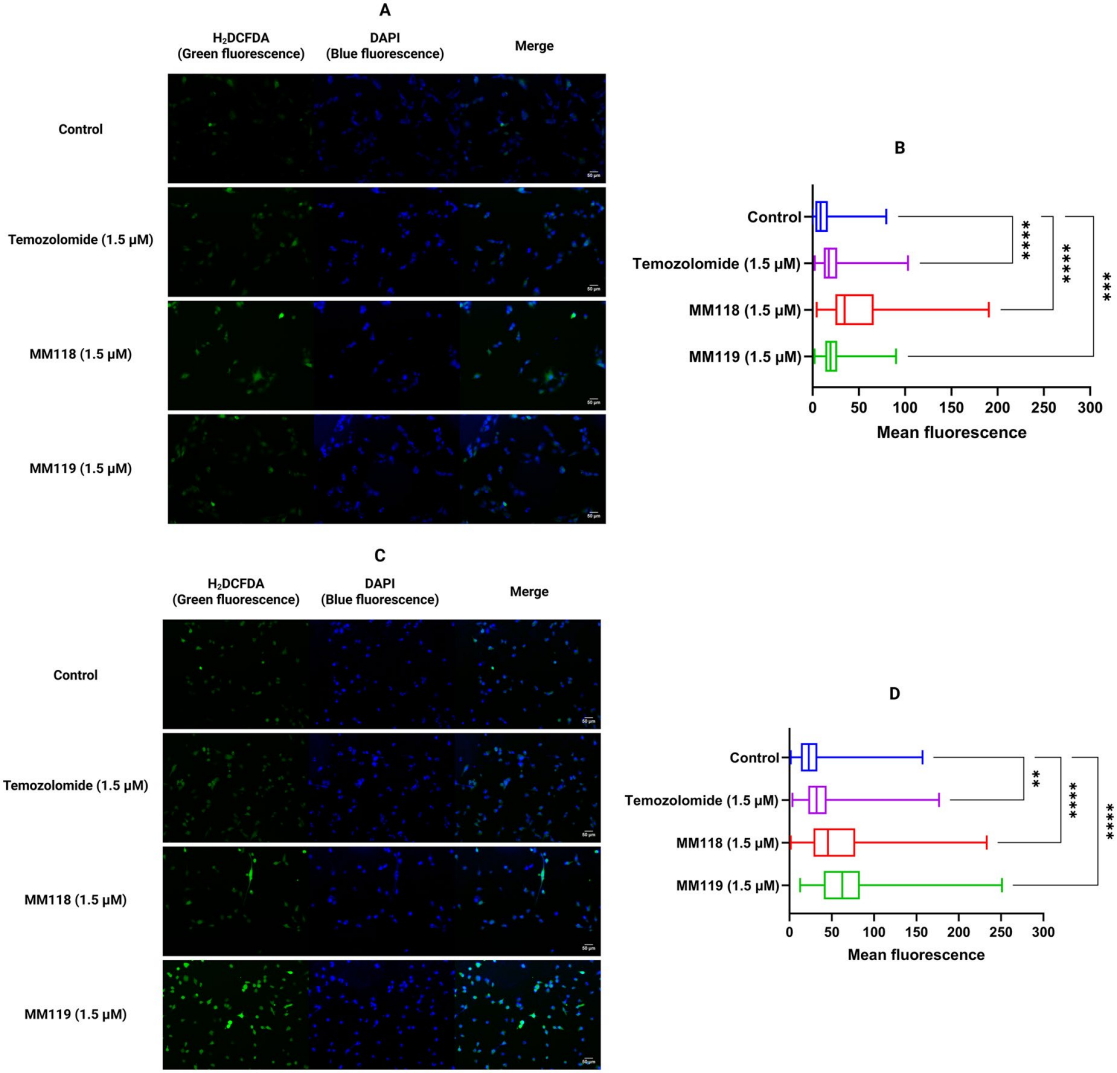
Results of H2DCFDA and DAPI double staining of glioblastoma cell lines after 30 min of compound treatment. Representative fluorescence microscope images are shown (A) - A172, and (C) U-87 MG cell lines. The graphs show the minimum, maximum and average fluorescence of each sample tested (B) - A172, and (D) U-87 MG. ANOVA tests were used to show differences between control and compound-treated cells. *N* > 100; **P ≤ 0.01; ****P ≤ 0.0001.

We performed double staining with DAPI, which stains nuclear genetic material (blue fluorescence), and H2DCFDA, an indicator of oxidative stress (green fluorescence). This study allowed us to evaluate the ability of **MM118** and **MM119** to activate reactive oxygen species (ROS) in A172 and U-87 MG glial cells. In A172 cells, an increase in mean green fluorescence was observed for both compounds, with **MM118** showing higher activity. In contrast, the U-87 MG cell line was more sensitive to the compounds tested and the mean fluorescence obtained for **MM118** and **MM119** was comparable (Figure 9). The results indicate that both compounds effectively induce oxidative stress in the cell lines tested.

### MM118 and MM119 cause activation of pyroptosis in glioblastoma cell lines

**Figure 10.**
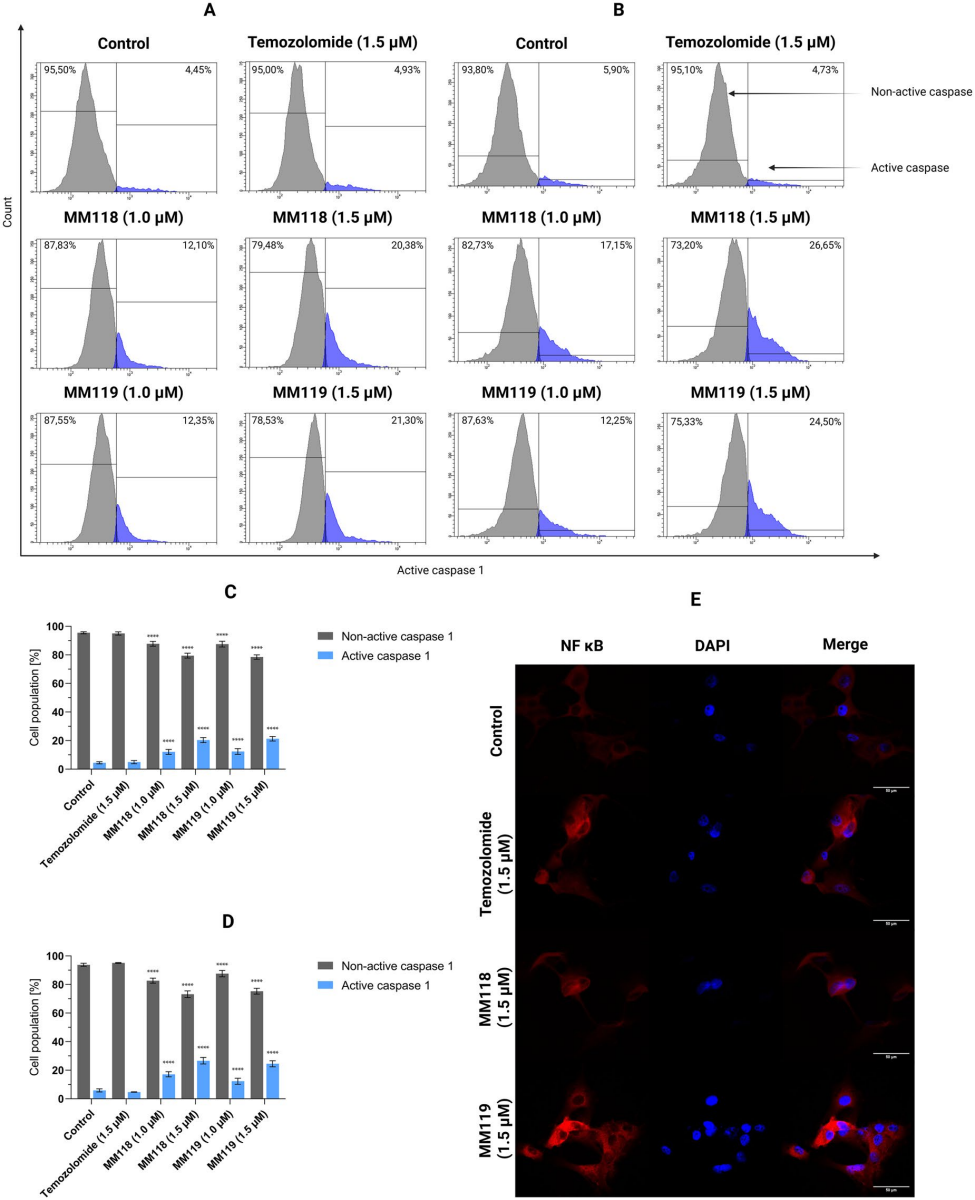
Results of flow cytometry analysis of caspase 1 activation of A172 (**A**) and U-87 MG (**B**) glioblastoma cells after 24 h of incubation with compounds. Graphs visualise the cytograms from the compound analysis A172 (**C**) and U-87 MG (**D**). Means ± SD are shown with *N* = 4. ANOVA tests were used to demonstrate differences between cells treated with compounds and control. ****P ≤ 0.0001 (**E**) Confocal microscope images with colocalization of NF-κB (red) with the nucleus (DAPI, blue) under treatment of TMZ, MM118 and MM119 or not (control). *In vivo* evaluation of compounds MM118 and MM119. The confocal images were acquired using a 63x oil immersion objective, *N* = 3.

Given the activation of oxidative stress and the occurrence of apoptotic cell death, we decided to verify whether other cell death pathways are triggered. The level of pyroptosis induction of the newly synthesised compounds **MM118** and **MM119** was determined by assessing the activation of caspase 1. This method facilitates the identification of cells undergoing pyroptosis by detecting the active form of caspase 1, which is a pivotal mediator in the pyroptotic pathway. The tested compounds were analysed on human A172 and U-87 MG glioblastoma multiforme cell lines using concentrations of 1 μM and 1.5 μM for a period of 24 h. In the case of the A172 line, the compound **MM118** at a concentration of 1 μM induced caspase 1 activation in 12.10 ± 1.69% of the cell population, while at a concentration of 1.5 μM this percentage increased to 20.38 ± 1.75%. Analogous relationships were observed in the U-87 MG line, where caspase 1 activation was 17.15 ± 1.81% and 26.65 ± 2.28%, respectively. Conversely, for compound **MM119** in line A172, caspase 1 activation was 12.35 ± 2.00% at a concentration of 1 μM and 21.30 ± 1.57% at a concentration of 1.5 μM. For the U-87 MG line, the values were 12.25 ± 2.16% and 24.50 ± 2.11%, respectively. For TMZ, no significant differences from the control sample were observed in any of the cell lines tested (Figure 10 A-D).

NF-κB, a crucial mediator of the inflammatory response, is a possible transcriptional regulator of caspase-1. Under resting conditions, the NF-κB/IκB complex remains in the cytoplasm, while upon activation, NF-κB translocates into the nucleus. Figure 10(E) shows that in case of all treatment conditions- whether MM118, MM119, or TMZ- NF-κB translocates to the cell nucleus.

**Figure 11.**
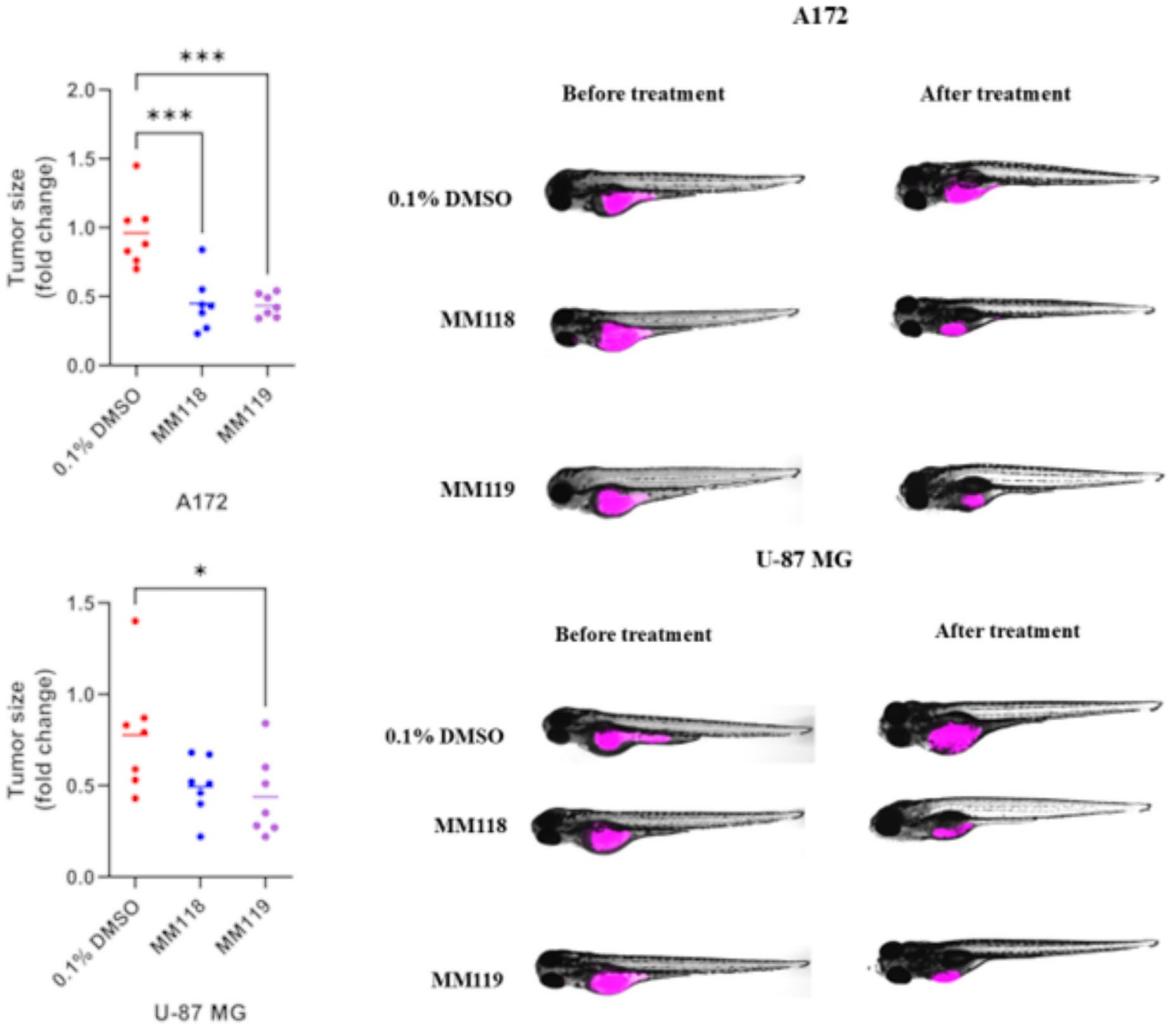
Effect of **MM118** and **MM119** treatment *in vivo* models. A) Zebrafish xenografts with U-87MG and A172 cell lines stained with Vybrant Did before and after **MM118** (10 mM) and **MM119** (10 mM) treatment. As a control, we used 0.1% DMSO. Representative pictures of 0 and 2 dpi (days post-injection) xenografts. B) Tumour size (changes of tumour area in each fish separately, *n* = 7) shown as fold change of the fish before and after treatment. Data were analysed using one-way analysis of variance (ANOVA) and Tukey’s *post hoc* test for comparisons (*p ≤ 0.05; ***p ≤ 0.001 was considered statistically significant).

The anti-tumour potential of **MM118** and **MM119** has been studied using *in vivo* model. Drugs were added to the 3-dpf fish embryo. Glioblastoma xenografts were monitored for next 2 days every 24 h. Compared to the control (xenografts treated by 0.1% DMSO), a decrease in tumour size was observed, in both **MM118** and **MM119** (Figure 11(A,B)).

In the case of the A172 glioblastoma cell line, the average tumour size reduction in zebrafish xenografts was observed to be 55% for **MM118** and 56% **MM119**, compared to only 4% decrease of tumour sizes in the control group with 0.1% DMSO. Similarly, for the U-87 MG, the tumour size reduction was 51% for **MM118** and 56% for **MM119**, whereas the control group with 0.1% DMSO showed a reduction of 22%.

## Materials and methods

### Synthesis of compounds MM118 and MM119

#### General

Melting points of the synthesised compounds were measured on a Mel-Temp Apparatus, and are reported without correction.^1^H (400 MHz) and ^13^C NMR (100 MHz) NMR spectra were obtained on a Varian spectrometer. Chemical shifts are expressed in parts per million (ppm) relative to tetramethylsilane (TMS) as an internal standard. Molecular weights of the synthesised compounds were determined by electrospray ionisation mass spectrometry (ESI-MS) with an Agilent 6538 UHD Accurate Mass Q-TOF LC/MS system. Elemental analyses deviated by no more than ±0.4% from the calculated values.

### Cell culture

Human glioblastoma cell lines (A172 CRL-1620^™^, U-87 MG HTB-14^™^, U-118 MG HTB-15^™^, T98G CRL-1690^™^) and the mouse astrocyte cell line (C8-D1A CRL-2541^™^) were obtained from the American Type Culture Collection (ATCC, Manassas, VA, USA). The cell lines were cultured in complete medium: DMEM (Corning, Kennebunk, ME, USA) for C8-D1A and T98G, and EMEM (ATCC, Manassas, VA, USA) for A172, U-87 MG, U118MG. The media were supplemented with 10% foetal bovine serum (Eurx, Gdansk, Poland) and 1% penicillin-streptomycin solution (Corning, Kennebunk, ME, USA). Cells were cultured in cell culture flasks (Sarstedt, Numbrecht, Germany) at 37 °C in 5% CO_2_. For experiments, cells were washed with phosphate-buffered saline without calcium and magnesium (Corning, Kennebunk, ME, USA) and treated with 0.05% trypsin and 0.02% EDTA (Corning, Kennebunk, ME, USA). Cells were counted using a Sceptre 3.0 cell counter (Merck Millipore, Burlington, MA, USA) prior to seeding into plates. For 6-well plates, 5 x 10^5^ cells/mL were seeded, while for 96-well plates (Sarstedt, Numbrecht, Germany), 1 x 10^5^ cells/mL were seeded in 100 μL of the medium.

### Evaluation of cytotoxicity of novel compounds by MTT assay

The cell lines were seeded in 96-well plates and cultured according to the methodology described in the previous section. Cells were then treated with the novel derivatives **MM118** and **MM119** and the reference compound temozolomide (**TMZ**) (Sigma-Aldrich, St. Louis, MO, USA) at concentrations of 0.1, 0.25, 0.5, 1.0, 2.5, 5.0, and 10.0 μM for 24 h. The MTT assay was performed using a solution of 3–(4,5-dimethyl-2-thiazolyl)-2,5-diphenyl-2H-tetrazolium bromide (Sigma-Aldrich, St. Louis, MO, USA) at a concentration of 5 mg/mL in phosphate-buffered saline without calcium and magnesium (Corning, Kennebunk, ME, USA). After 24 h incubation with test compounds, 10 μL MTT solution was added to each well, and cells were incubated for another 2 h at 37 °C. The cells were then lysed in 100 μL lysis buffer (25 mM HCl, 2% acetic acid, 3% DMF, and 5% SDS, adjusted to pH 4.7). Absorbance was measured at 570 nm using an Absorbance 96 microplate reader (Byonoy GmbH, Hamburg, Germany).

### Evaluation of cytotoxicity by LDH assay

Analogous to the procedure described in the previous section, A172 and U-87 MG cells were cultured and plated in 96-well plates. The LDH-Cytotoxicity Assay Kit II (ab65393) (Abcam, Cambridge, UK) was used to assess cytotoxicity according to the manufacturer’s protocol. The test compounds **MM118** and **MM119** and the reference compound temozolomide (**TMZ**) were added to the cells at a concentration of 1.5 μM. After 24 h of exposure, the medium was collected, centrifuged and 10 μL of each sample was transferred to a new 96-well plate. In the next step, 100 μL of LDH Reaction Mix was added to each well and incubated for 30 min at room temperature. Absorbance was measured at 450 nm using an Absorbance 96 microplate reader (Byonoy GmbH, Hamburg, Germany). Cytotoxicity was calculated according to the formula provided by the manufacturer: **Cytotoxicity (%)** = ((Test Sample – Low Control)/(High Control – Low Control)) X 100, where Low Control - control without added compound, High Control with added 10 µL Lysis Buffer II.

### Annexin V-FITC and propidium iodide (PI) apoptosis analysis

The impact of apoptosis induction by the tested compounds at concentrations of 1 μM and 1.5 μM, as well as by temozolomide at 1.5 μM, was evaluated through annexin V-FITC and propidium iodide (PI) staining with the Apoptosis Detection Kit II (BD Biosciences, San Diego, CA, USA). The glioblastoma cell lines A172 and U-87 MG were then deprived of medium and washed several times with cold phosphate-buffered saline (PBS) solution. The cell suspension was then prepared in the binding buffer provided in the kit at a concentration of 1 × 10^6^ cells/mL. From each sample, 100 μL of the cell suspension was transferred to tubes and subsequently treated with 5 μL of Annexin V-FITC and 5 μL of propidium iodide (PI). The tubes were then incubated for 15 min at room temperature in the dark. Following the incubation period, 400 μL of binding buffer was added to each sample, after which the samples were immediately analysed using a BD FACSCanto II flow cytometer (Becton Dickinson Biosciences, San Jose, CA, USA), with 10,000 events recorded per sample. The results were then analysed using FACSDiva Software Version 6.1.3 (BD Biosciences Systems, San Jose, CA, USA).

### Effect on mitochondrial membrane potential (ΔΨ_m_)

JC-1 (1,1′,3,3′-Tetraethyl-5,5′,6,6′-tetrachloroimidacarbocyanine iodide) (BD Biosciences, San Diego, CA, USA), a lipophilic fluorochrome, was used to evaluate changes in mitochondrial membrane potential. After treatment, cancer cells at a concentration of 1 × 10^6^ cells/mL were collected and rinsed twice with PBS. Then, 500 μL JC-1 working solution was added to each sample according to the manufacturer’s protocol. The samples were then incubated for 15 min at 37 °C in a CO_2_ incubator. After incubation, samples were washed twice with an assay buffer, and 500 μL of each sample was transferred to a BD FACSCanto II flow cytometer (Becton Dickinson Biosciences, San Jose, CA, USA) for analysis, with 10,000 events recorded per sample. Data were analysed using FACSDiva software version 6.1.3 (BD Biosciences Systems, San Jose, CA, USA).

### Impact of new compounds on caspase 1, 3/7, 8 and 9 activity

The activity of caspase 1, caspase 3/7, caspase 8, and caspase 9 was evaluated through the utilisation of FAM FLICA Caspase Assays (ImmunoChemistry Technologies, Bloomington, MN, USA), in accordance with the established protocols. Following treatment with the compounds, the cells were collected at a concentration of 1 × 10^6^ cells/mL, washed twice with cold PBS, and resuspended in 290 μL of PBS. The subsequent step involved the addition of 10 μL of 30X FLICA solution, followed by incubation at 37 °C in the absence of light for one hour. Then, 2 ml of Apoptosis Wash Buffer was added, and the samples were washed twice. A 300 μL aliquot of each sample was transferred to a BD FACSCanto II flow cytometer (Becton Dickinson Biosciences, San Jose, CA, USA), where 10,000 events per sample were recorded. The resulting data was then analysed using FACSDiva software, version 6.1.3 (BD Biosciences Systems, San Jose, CA, USA).

### Reactive oxygen species assessment

The impact of the tested compounds on reactive oxygen species (ROS) production was assessed. Cells were seeded in 6-well culture plates at a density of 1 × 10^5^ cells per well in 1 ml of medium. Following a 30-min treatment with the compounds, cells were washed with phosphate-buffered saline (PBS). Subsequently, double staining was performed using 20 µM 2′,7′-dichlorodihydrofluorescein diacetate (H_2_DCFDA) (obtained from Thermo Fisher Scientific, Eugene, OR, USA). This colourless reagent is hydrolysed by intracellular esterases and subsequently oxidised to the 2′,7′-dichlorofluorescein (DCF), and 10 µg/mL of 4′,6-diamidino-2-phenylindole (DAPI) (Sigma-Aldrich, St. Louis, MO, USA) was used to stain nuclear DNA. Fluorescence was observed using a Nikon ECLIPSE Ti fluorescence microscope (Nikon, Tokyo, Japan). ImageJ software (Bethesda, MD, USA) was used for data analysis, with DCF fluorescence intensity measured relative to the control. A minimum of 100 cells per sample were analysed.

### NF-kB staining

Location of NF-kB was evaluated by immunofluorescence staining. Cells were cultured on sterile coverslips placed in 6-well plates and treated with compounds MM118, MM119, and TMZ at a concentration of 1.5 μM. After 24 h of treatment, cells were fixed with 4% paraformaldehyde for 15 min at room temperature, then permeabilized with 0.1% Triton X-100 in PBS for 10 min. Following permeabilization, cells were blocked with 2% BSA (bovine serum albumin; Cell Signalling Technology, Danvers, MA, USA) in PBS for 30 min to prevent non-specific binding. Subsequently, cells were incubated overnight at 4 °C with a NF-KB antibody directly conjugated with Alexa Fluor 647 (#D14E12; Cell Signalling Technology, Danvers, MA, USA). The next day, after washing with PBS, nuclei were counterstained with DAPI (4′,6-diamidino-2-phenylindole; Sigma-Aldrich, St. Louis, MO, USA) for 5 min at room temperature. Coverslips were mounted onto glass slides using an antifade mounting medium and imaged using a Stellaris 5 confocal microscope (Leica Microsystems, GmbH, Wetzlar, Germany). Images were analysed using ImageJ software (Bethesda, MD, USA).

### Zebrafish handling

In our study, we used wild-type AB zebrafish embryos obtained from the certified Experimental Medicine Centre (Lublin, Poland). Adult zebrafish were raised at 28.5 °C under 14 h of light and 10 h of the dark cycle and fed in accordance with the guidelines established by the Research Animals Department of the RSPCA. Fertilised eggs were obtained by natural spawning; healthy clutches were processed for study or propagated for line maintenance. Embryos were maintained in the E3 medium (5 mM NaCl, 0.33 mM MgCl2, 0.33 mM CaCl2, 0.17 mM KCl; pH 7.2) at 28.5 °C. The use of animals in scientific research in Europe is governed by the Directive 2010/63/EU of 22 September 2010. According to this directive, experiments involving zebrafish embryos up to 5 days post-fertilization (dpf) do not require approval from an ethics committee, as they are not considered capable of independent feeding and thus fall outside the definition of protected animals. All procedures in this study were performed on embryos up to 5 dpf.

### Creating zebrafish xenografts

A172 and U-87MG cell lines were labelled with Vybrant Did (Invitrogen, Waltham, MA, USA) according to the manufacturer’s protocol. Stained cells were re-suspended in Dulbecco’s modified Eagle’s medium (DMEM) at the final concentration of 1 × 10^7^ cells/mL. 24 h post-fertilisation (hpf) zebrafish were manually dechorionated. 48 hpf zebrafish were anaesthetised by placing in 0.04 mg/mL ethyl 3-aminobenzoate methanesulfonate tricaine. Approximately 300 labelled cells were injected into the interior yolk space of each embryos using an electronically regulated air-pressure microinjector (Narishige IM-300 Microinjector, Tokyo, Japan). After injection, the zebrafish were washed once with fish water and transferred to a 24-well plate one fish per one well containing 2 ml of E3 medium water and incubated at 32 °C^17^.

### Xenografts drugs treatment

MM118 and MM119 stock were dissolved in DMSO. A172 and U-87MG-xenografts (72 hpf) were incubated with M118 (10 µM) and M119 (10 µM) for 48 h in 24 well plate one fish per one well. The final volume of the medium in each well was 2 ml. DMSO was used as a drug solvent. The final concentration of DMSO in the wells did not exceed the damaging concentration of above 0.1%. The mock control embryos were incubated in embryo medium in the presence of 0.1% DMSO.

### Xenografts imaging

Xenograft imaging has been performed before (48 hpf) and after (120 hpf) drugs treatment using an EVOS M5000 Imaging System with the filter Cy5 (excitation: 628 nm; emission: 692 nm). The zebrafish were anaesthetised by 0.04 mg/mL ethyl 3-aminobenzoate methanesulfonate tricaine before imaging. After imaging, fish were washed from tricaine twice with E3. Tumour sizes were analysed as the area of labelled tumour cells according Ali et al.^18^ using EVOS software.

### Statistical analysis

All statistical analyses were conducted using GraphPad Prism Version 6.0 (San Diego, CA, USA). The results are presented as the mean ± standard deviation (SD). The analysis of variance (ANOVA) and Tukey tests were employed to demonstrate the differences between the samples that were exposed to varying concentrations of the test compounds. A statistically significant difference was defined as follows: * *P* ≤ 0.05; ** *P* ≤ 0.01; *** *P* ≤ 0.001; **** *P* ≤ 0.0001; ns - not statistically significant.

## Discussion

Triazines have already been utilised in cancer treatment, as exemplified by the clinically approved alkylating agent temozolomide, our reference compound^19^. Given this established therapeutic relevance, we selected compounds from the MM series—specifically the isomers MM118 and MM119—for evaluation in a glioblastoma model, leveraging the potential of this class in the treatment of malignant brain tumours.

Earlier studies on MM compounds demonstrated significant cytotoxicity against various cancer cell lines^20–22^. In our study, the cytotoxic effects of MM118 and MM119 were assessed using MTT and LDH assays on five cell lines: A172, U-87 MG, U-118 MG, T98G, and C8-D1A. Based on these results, we selected the A172 and U-87 MG cell lines for further investigation, as they represent distinct glioblastoma models. A172 cells are known for their high migratory potential and ability to infiltrate surrounding brain tissue, making them a suitable model for studying glioblastoma invasion mechanisms^23^. These cells exhibit elevated expression of genes associated with epithelial-to-mesenchymal transition, which is often linked to increased tumour aggressiveness and resistance to therapy^24^. In contrast, U-87 MG cells are more proliferative but less invasive, representing a glioblastoma model with well-defined tumour boundaries^25^. The differences between these two models allowed us a comprehensive evaluation of the anti-glioblastoma potential of MM118 and MM119 compounds in both invasive and proliferative tumour environments.

To further explore the effects of MM118 and MM119, we examined their ability to induce cell death using Annexin V/Propidium Iodide (AV/PI) staining. Our findings confirmed that these compounds, at a concentration of 1.5 µM, effectively stimulated cell death. Consequently, we proceeded to analyse mitochondrial membrane potential and the activation of caspases 3/7, 8, and 9. The results demonstrated caspase activation, indicating the involvement of cell death pathways. Previous studies have similarly reported cell death induction and decreased mitochondrial membrane potential^20^.

Additionally, MM129, MM130, and MM131 have been shown to induce DNA damage, including both single-strand breaks (SSBs) and double-strand breaks (DSBs), as evidenced by comet assays^21^. DNA damage serves as an indicator of genotoxicity, often resulting from oxidative stress and the presence of free radicals, which contribute to DNA fragmentation^26^. Given this background, we sought to determine whether MM118 and MM119 could induce oxidative stress. Using H_2_DCFDA staining, we confirmed that these compounds indeed triggered oxidative stress.

Given the oxidative stress induced by compounds MM118 and MM119, we decided to investigate caspase 1 activity, as its presence is a key indicator of pyroptosis, an inflammatory form of cell death that differs from classical apoptosis in both mechanism and biological consequences^27^. Unlike apoptosis, which is a tightly regulated process that does not elicit a strong immune response, pyroptosis is characterised by the rapid disintegration of the cell, the release of cytoplasmic contents into the extracellular space, and the activation of a cascade of pro-inflammatory factors^28^^,^^29^. Central to this process is the inflammasome, a multiprotein sensing complex that, upon detecting danger signals such as cell damage or the presence of pathogens, activates caspase 1. This, in turn, leads to the maturation and release of pro-inflammatory cytokines, which can intensify the inflammatory response, affecting the tissue microenvironment and further recruitment of immune cells^30^^,^^31^. In the context of the action of compounds MM118 and MM119, the analysis of caspase 1 activity provided important information on their potential immunomodulatory and anticancer properties. NF-κB, a well-known transcription factor involved in the regulation of the inflammatory response, is a prime candidate as a transcriptional regulator of caspase-1. In the resting state, NF-κB dimers are trapped in the cytoplasm through interaction with the IκB protein. Upon activation, NF-κB dissociates from the inhibitor and moves into the cell nucleus, where it controls the expression of numerous genes associated with the immune response^32^. Therefore, we conducted a qualitative analysis of NF-κB localisation after treatment with MM118, MM119, and TMZ. The confocal microscope images show the displacement of NF-κB into the cell nucleus for both compounds MM118, MM119, and the reference compound TMZ.

The tumour reduction observed in the control group of zebrafish may be linked to the activation of the immune system, which could have contributed to a modest antitumor effect even in the absence of targeted therapeutic agents. The first immune response in zebrafish occurs as early as 12 h post-fertilization (hpf), marked by the activation and migration of neutrophils^33^. These innate immune cells are rapidly mobilised to sites of injury or foreign presence, including tumour xenografts, where they can exert cytotoxic and pro-inflammatory effects. Furthermore, *in vitro* studies indicate that the tested compounds may have played a key role in enhancing the immune response by inducing pyroptosis—an inflammatory form of programmed cell death. This process may amplify the natural immune response against tumour cells, creating a more hostile microenvironment for tumour growth. Consequently, the observed tumour reduction may be attributable not only to the intrinsic immune activity of zebrafish but also to the immune-modulating effects of the tested compounds, which enhance the body’s capacity to target and eliminate cancer cells.

## Conclusions

MM118 and MM119 exhibit potent cytotoxic effects against glioblastoma multiforme cells, as confirmed by *in vitro* assays. Their mechanism of action includes induction of apoptosis through activation of caspases and disruption of the mitochondrial membrane potential. In addition, these compounds induce oxidative stress, which may contribute to their cytotoxic effect. Activation of caspase-1 suggests their potential role in the induction of pyroptosis, which may lead to additional immunomodulatory effects. In an in vivo model on zebrafish, tumour shrinkage was observed, which may be due to both the direct action of the compounds and their effect on the body’s immune response. These results indicate that MM118 and MM119 may be promising candidates for further research into the therapy of glioblastoma multiforme, combining cytotoxic and immunomodulatory effects.

## Data Availability

The datasets generated during and/or analysed during the current study are not publicly available but are available from the corresponding author on reasonable request.
